# Oseltamivir Prescription and Regulatory Actions Vis-à-Vis Abnormal Behavior Risk in Japan: Drug Utilization Study Using a Nationwide Pharmacy Database

**DOI:** 10.1371/journal.pone.0028483

**Published:** 2011-12-06

**Authors:** Hisashi Urushihara, Yuko Doi, Masaru Arai, Toshiyuki Matsunaga, Yosuke Fujii, Naoko Iino, Takashi Kawamura, Koji Kawakami

**Affiliations:** 1 Department of Pharmacoepidemiology, Graduate School of Medicine and Public Health, Kyoto University, Kyoto, Japan; 2 Ain Pharmaciez Inc., Tokyo, Japan; 3 Kraft Inc., Tokyo, Japan; 4 Department of Clinical Research and Informatics, Research Institute National Center for Global Health and Medicine, Tokyo, Japan; 5 Department of Preventive Service, Graduate School of Medicine and Public Health, Kyoto University, Kyoto, Japan; University of Liverpool, United Kingdom

## Abstract

**Background:**

In March 2007, a regulatory advisory was issued in Japan to restrict oseltamivir use in children aged 10-19 years because of safety concerns over abnormal behavior. The effectiveness and validity of regulatory risk minimization actions remain to be reviewed, despite their significant public health implications. To assess the impact of the regulatory actions on prescribing practices and safety reporting.

**Methodoloy/Prinicpal Findings:**

In this retrospective review of a nationwide pharmacy database, we analyzed 100,344 dispensation records for oseltamivir and zanamivir for the period from November 2006 to March 2009. The time trend in dispensations for these antiviral agents was presented before and after the regulatory actions, contrasted with intensity of media coverage and the numbers of spontaneous adverse reaction reports with regard to antivirals.

The 2007 regulatory actions, together with its intense media coverage, reduced oseltamivir dispensation in targeted patients in fiscal year 2008 to 20.4% of that in fiscal year 2006, although influenza activities were comparable between these fiscal years. In contrast, zanamivir dispensation increased approximately nine-fold across all age groups. The number of abnormal behavior reports associated with oseltamivir in children aged 10-19 years decreased from fiscal year 2006 to 2008 (24 to 9 cases); this decline was offset by the increased number of reports of abnormal behavior in children under age 10 (12 to 28 cases). The number of reports associated with zanamivir increased in proportion to increased dispensation of this drug (11 to 114 cases).

**Conclusions/Significance:**

The 2007 actions effectively reduced oseltamivir prescriptions and the number of reports of abnormal behavior in the targeted group. The observed increase in abnormal behavior reports in oseltamivir patients under age 10 and in zanamivir patients suggests that these patient groups may also be at risk, calling into question the validity of the current discrimination by age and agent (Abstract translation is available in Japanese: Appendix S1).

## Introduction

The neuraminidase inhibitors oseltamivir and zanamivir are the most frequently used antivirals in the treatment of influenza. Both have been proved effective in the treatment and prevention of influenza, with good tolerability from children to elderly adults [1], [2]. Following the launch of oseltamivir on January 2001, Japan accounted for 75% of global consumption, or 34 million patients as of March 2007 [3]. However, post-marketing experience raised public and regulatory concerns over the safety profile of this drug [4]; two reported cases of falls associated with abnormal behavior in teenagers using oseltamivir eventually triggered regulatory actions in March 2007, namely a Dear Doctor Letter (DDL) and package insert (PI) revision recommending against oseltamivir use in children aged 10–19 years. The Ministry of Health, Welfare and Labour (MHLW) took this decision based on the precautionary principle without ample demonstration of causality of abnormal behavior [5].

In contrast, with regard to zanamivir, a drug in the same class as oseltamivir, the only change in the PI has been the addition of possible neuropsychiatric symptoms in the section titled “important precautions” made in January 2008 [6]. The oseltamivir-associated risk of neuropsychiatric disorders has been controversial [7], [8], and no other regulatory agency has taken such definitive countermeasures to date [9], [10]. Moreover, the effectiveness and validity of these 2007 regulatory actions remain to be reviewed, despite their significant public health implications.

Here, to assess the effectiveness of the 2007 regulatory actions in minimizing risk of oseltamivir-associated abnormal behaviors, we examined drug dispensation records sourced from nationwide pharmacy chains as an index of trends in the use of antivirals for influenza treatment in the Japanese prescribing community, and media coverage of these antivirals. We also reviewed the national database of spontaneous adverse reaction reports in relation to these drugs.

## Methods

### Data collection

This study is a time-trend study of antiviral drug utilization using a nationwide pharmacy claim database. The database consisted of claim and dispensation records obtained from the largest and third largest pharmacy chains in Japan (Ain Pharmaciez Inc. and Kraft Inc.), which incorporated the electronic records of prescriptions filled at 433 community pharmacies located around Japan. The database contains data on approximately 10 million outpatient prescriptions per year in total, accounting for approximately 1.4% of total yearly outpatient prescriptions in Japan [11], corresponding to a total patient coverage of approximately 5.5 million. The age distribution of patients in the database is well matched to national patient statistics [12]. These two pharmacy chains primarily cover the most-populated middle and eastern areas of Japan, from Kansai to Hokkaido, with only minor representation in southwest Japan, including the Chugoku, Shikoku, and Kyushu areas.

Dispensations of oseltamivir or zanamivir were examined to investigate the impact of regulatory actions on prescribing trends. Amantadines were excluded, owing to their infrequent use for influenza in Japan. We studied drug dispensation records for the prescriptions written between November 1, 2006, and March 31, 2009, a period which incorporated the DDL and subsequent PI revision of oseltamivir, dated March 20, 2007. De-identified dispensation records included an anonymized patient identifier, age, gender, name, and code of the medical institution, specialty, code of the dispensing pharmacy, date of prescription, code and brand name of the drug, dosing days, and dosage. Antiviral prescriptions with a dosage frequency of twice a day or more for therapeutic purposes were eligible for analysis. Prescriptions with a “once a day” dosage for prevention and those with missing dosage information were excluded from analysis. A single dispensation record for either oseltamivir, zanamivir or both were counted as a single episode of influenza unless antiviral dispensing was repeated within the next five days under the same patient identifier. When there were consecutive dispensation records of antivirals within five days for the same patient, total dosing days of a single treatment course for a single influenza case were calculated by combining these records on the basis that the standard dosing practice for the treatment of influenza is five days [13], [14].

Weekly influenza activity is reported by the National Epidemiological Surveillance of Infectious Diseases (NESID) based on surveillance of approximately 5,000 sentinel hospitals and clinics throughout Japan [15]. We used the mean incidences of clinical influenza per sentinel site as an index of weekly influenza activity at the national level for analysis.

Media coverage of oseltamivir and zanamivir was evaluated using the G-Search database (G-Search Limited) [16]. The database was searched for articles containing the keywords “Tamiflu” or “Oseltamivir,” and “Relenza” or “Zanamivir” in the four major national and eight major regional newspapers in Japan during the study period. The number of articles per month was counted separately for oseltamivir and zanamivir.

Regulatory reporting on suspected adverse reactions associated with oseltamivir and zanamivir were identified in the national database for spontaneous case reports of suspected adverse reaction reported to the Pharmaceuticals and Medical Devices Agency (PMDA) [17]. Each report in the PMDA database contains an event term reported by the healthcare professional, the suspected drug's name, the reporter's opinion on causality with the suspected drug, outcome of the event, the fiscal year of the report, and patient's age decile. Reported events are coded in accordance with the Medical Dictionary for Regulatory Activities coding system, and summarized by a Lower Level Term and a System Organ Class [18].

### Ethics

Because the pharmacy claim data investigated in the present study were retrieved from the automated electronic database and de-identified before provision to the study group, the study was exempt from obtaining informed consent from individual patients according to the local ethical guideline for epidemiological research. This study and the waiver of informed consent were approved by the Kyoto University Graduate School and Faculty of Medicine, Ethics Committee (No. E-775).

### Statistical analysis

Our analyses are descriptive in nature. The number of influenza cases treated with oseltamivir or zanamivir (or both) was aggregated for intervals appropriate for the intended analyses. To validate the reliability of drug dispensation data as representative of national medication trends in influenza treatment, the number of cases treated with oseltamivir or zanamivir weekly was correlated using the Pearson correlation coefficient with weekly national influenza activity reported by the NESID. The impact of the regulatory actions and media coverage about antivirals on prescribing trends of oseltamivir and zanamivir were evaluated using the monthly or yearly number of dispensations stratified by age groups with a 10-year interval. The numbers of yearly dispensations were summarized for each fiscal year starting on April 1 and ending on the following March 31 because of the following reasons: the yearly number of dispensed cases was compared before and after the regulatory actions taken at the end of March 2007, which coincided with the end of fiscal year 2006. Further, the PMDA spontaneous reporting database only includes data of fiscal year when each case was reported, not the actual onset date; therefore, these cases were summarized by the fiscal year of reports, to be contrasted with the annual antiviral dispensations. However, the number of dispensed cases for fiscal year 2006 was calculated from records between November 1, 2006, and March 31, 2007, due to limited data availability of pharmacy records. Thus, the reporting year may not necessarily reflect the actual year when the event occurred. The indices for yearly trends of adverse reaction reporting were calculated by dividing the yearly numbers of spontaneous case reports by the numbers of influenza cases dispensed each antiviral at the study pharmacies. A chi-square test for independence was used with a two-tailed significance level of 0.05. Statistical analyses were performed with SPSS 18 for Windows (SPSS Inc., Chicago, IL, USA).

## Results

**Figure 1:**
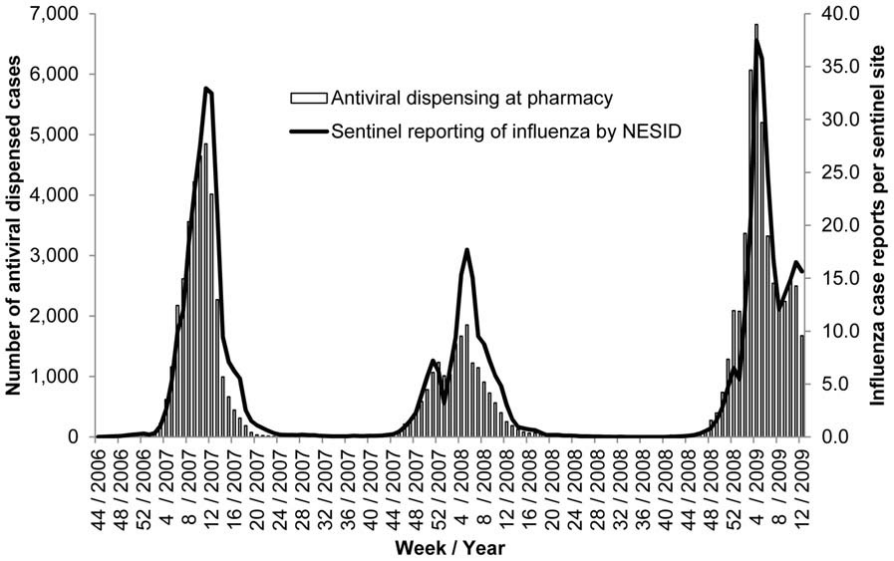
Correlation between antiviral dispensation at community pharmacies and sentinel reporting of influenza activities in Japan. NESID, the National Epidemiological Surveillance of Infectious Diseases.

Between November 1, 2006, and March 31, 2009, a total 100,344 dispensation records for oseltamivir and zanamivir were analyzed. The 1,015 dispensation records for prevention or with missing dosage were excluded from analysis. After eliminating records for refills within a single treatment course, we identified 97,954 influenza cases (males, 50.8%) treated with the antivirals, of which 77,695 (males, 51.1%) were treated with oseltamivir, with a median age of 15 years (range, 0–102); and 20,382 cases (males, 49.9%) treated with zanamivir, with a median age of 13 years (range, 0–96). The weekly number of antiviral treatment cases was highly correlated with the NESID influenza activity index (r = 0.945, Figure 1) throughout the study period, indicating that the antiviral dispensation records in the pharmacy claim database effectively represented the national trend in influenza antiviral treatment.

### Effect of risk minimization actions on antiviral dispensations and media coverage

**Figure 2:**
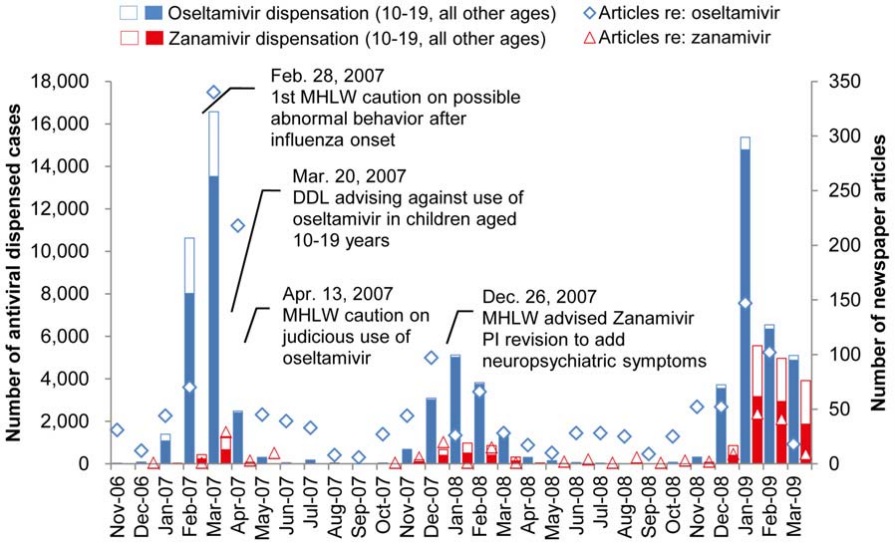
Antiviral dispensation at community pharmacies, related regulatory actions and media publicity in Japan. Open boxes at the top of each bar indicate the amount of dispensations to children aged 10–19 years. MHLW, the Ministry of Health, Welfare and Labour; DDL, Dear doctor letter; PI, package insert.

Several actions in response to safety concerns of oseltamivir and zanamivir in Japan were taken by the regulatory body and academic societies after multiple cases of fatal falls in teenagers were reported in February 2007 (Table S1). Oseltamivir dispensing to children aged 10–19 years during the period from November 2006 to March 2007, that is, before the March 2007 regulatory actions, accounted for 20.9% of the total (Table 1). Coverage of oseltamivir in major newspapers during this period mainly concerned the association of accidental falls with oseltamivir use, with the intensity of reportage peaking in the month of the regulatory advisory, issued on March 20, 2007 (Figure 2). In fiscal year 2007, the number of oseltamivir-dispensed cases was reduced to 60.1% across ages compared with the levels in fiscal year 2006, and dispensation to children aged 10–19 years accounted for only 2.3% of the total (p = 0.000, vs. fiscal year 2006). On comparison of the 2006/2007 and 2008/2009 seasons, which had comparable influenza activities (Figure 1), the total number of oseltamivir dispensations in fiscal year 2008 was almost equal to that in fiscal year 2006 (110.4%). However, the dispensation in children aged 10–19 years decreased to 20.4% of the fiscal year 2006 level, corresponding to 3.9% of the total in fiscal year 2008 (p = 0.000, vs. 2006). In contrast, dispensation of zanamivir increased approximately 8.8-fold in fiscal year 2008, including a proportional increase in children aged 10–19 years (855%). Yearly zanamivir dispensation to children aged 10-19 years accounted for around 45% of total dispensing throughout the study period (Table 1, p = 0.407 and 0.297 for fiscal years 2007 and 2008, vs. fiscal year 2006). Most media coverage on antivirals during fiscal year 2008 was related to preparations, such as national stock piling, for pandemic influenza.

**Table 1:** Yearly dispensation of antiviral agents by age groups at community pharmacies in Japan.

	Oseltamivir			Zanamivir		
Age group	FY2006 (%)†	FY2007 (%)	FY2008 (%)	FY2006 (%)†	FY2007 (%)	FY2008 (%)
0-9	10,039 (34.9)	7,471 (43.3)	15,702 (49.5)	383 (21.8)	740 (23.6)	3,501 (22.6)
10-19	5,991 (20.9)	389 (2.3)	1,221 (3.9)	822 (46.7)	1,507 (48.0)	7,032 (45.4)
20-29	3,401 (11.8)	2,705 (15.7)	4,136 (13.0)	174 (9.9)	316 (10.1)	1,291 (8.3)
30-39	3,732 (13.0)	3,230 (18.7)	4,729 (14.9)	180 (10.2)	289 (9.2)	1,713 (11.1)
40-49	2,421 (8.4)	1,791 (10.4)	3,031 (9.6)	96 (5.5)	161 (5.1)	1, 083 (7.0)
50-59	1,548 (5.4)	942 (5.5)	1,488 (4.7)	62 (3.5)	70 (2.2)	446 (2.9)
60-	1,592 (5.5)	732 (4.2)	1,404 (4.4)	42 (2.4)	59 (1.9)	415 (2.7)
Total	28,724	17,260	31,711	1,759	3,142	15,481

FY, fiscal year.

Values represent number of cases.

† Dispensations during each fiscal year represent the number of dispensations calculated from pharmacy dispensation records for a period starting on April 1 and ending on the following March 31, except for fiscal year 2006 calculated from records between November 1, 2006, and March 31, 2007, due to limited data availability of pharmacy records.

### Spontaneous adverse reaction reporting and dispensing trends

**Figure 3:**
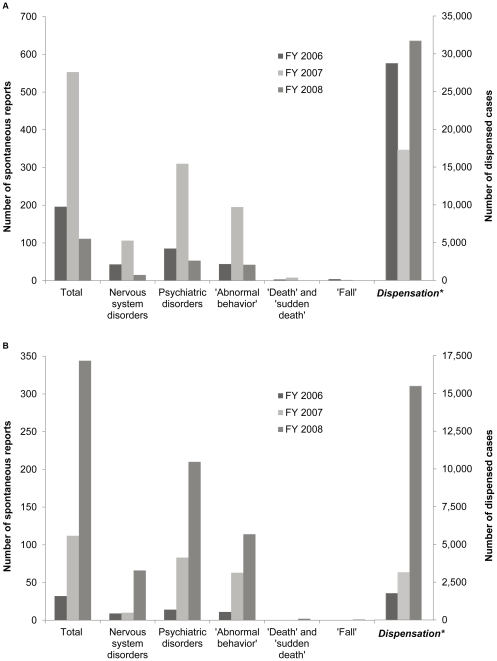
Adverse reactions reported for oseltamivir and zanamivir and antiviral dispensations. A. Oseltamivir, B. Zanamivir Single quotation denotes individual Lower Level Term of the MedDRA coding system, although no quotation indicates System Organ Class. FY, fiscal year starting on April 1 and ending on the following March 31. *The first bar for dispensation during fiscal year 2006 represents the number of dispensations calculated from pharmacy dispensation records between November 1, 2006, and March 31, 2007, due to limited data availability in the pharmacy claim database.

Suspected adverse reactions reported for oseltamivir and zanamivir throughout the study period were predominately psychiatric disorders, including abnormal behavior (Figure 3). The largest number of spontaneous case reports associated with oseltamivir occurred in fiscal year 2007, for all categories of the relevant event terms and classes, including eight cases of death and two of falls. This occurred despite that year being documented as having the lowest annual number of dispensations, indicating a high possibility that the preceding March 2007 regulatory advisory stimulated reporting of abnormal behavior associated with oseltamivir in fiscal year 2007 (Table 1, Figure 3A).

In fiscal year 2008, overall spontaneous reporting associated with oseltamivir decreased to below that in fiscal year 2006, whereas total dispensing increased up to the level of fiscal year 2006. The number of case reports of abnormal behavior in children aged 10-19 years decreased from 24 in fiscal year 2006 to 9 in fiscal year 2008 in parallel with the reduction in dispensation (Table 2). However, this decrease in abnormal behavior reports was offset by new cases arising in children aged less than 10 years (from 12 in fiscal year 2006 to 28 in fiscal year 2008) in parallel with increased dispensation to this age group in fiscal year 2008. Thus, the total number of abnormal behavior reports remained steady, from 44 in fiscal year 2006 to 42 in fiscal year 2008. No cases of death or falls were reported for oseltamivir in fiscal year 2008.

**Table 2:** Comparison of antiviral dispensation and abnormal behaviors reports between 2006/2007 and 2008/2009 seasons in childhood patients.

	Age group	Variables	FY2006†	FY2008	P values*
Oseltamivir	0-9	Dispensed cases	10,039	15,702	
		Abnormal behavior reports	12	28	
		Reporting trend adjusted for 1,000 dispensed case	1.20	1.78	0.244
	10-19	Dispensed cases	5,991	1,221	
		Abnormal behavior reports	24	9	
		Reporting trend adjusted for 1,000 dispensed case	4.01	7.37	0.114
Zanamivir	0-9	Dispensed cases	383	3,501	
		Abnormal behavior reports	5	35	
		Reporting trend adjusted for 1,000 dispensed case	13.05	10.00	0.578
	10-19	Dispensed cases	822	7,032	
		Abnormal behavior reports	6	78	
		Reporting trend adjusted for 1,000 dispensed case	7.30	11.09	0.322

FY, fiscal year.

† Fiscal year represents a period starting on April 1 and ending on March 31, although, the dispensations in fiscal year 2006 represent the numbers of dispensations calculated from records between November 1, 2006, and March 31, 2007, due to limited data availability of pharmacy records.

*P values of chi-square tests for comparison between fiscal years.

Further, to examine changes in abnormal behavior reporting for oseltamivir before and after the March 2007 regulatory advisory, the 2006/2007 and 2008/2009 seasons, which had comparable influenza activities and may therefore have been less affected by stimulated reporting, were compared. No significant differences were noted between fiscal years 2006 and 2008 in the reporting trends of abnormal behaviors after adjustment for the numbers of oseltamivir dispensations in children aged less than 10 years and 10-19 years (Table 2).

With regard to zanamivir, in contrast, increases in the number of spontaneous case reports for total adverse reactions (32 to 344 cases), nervous system disorders (9 to 66 cases), and psychiatric disorders (14 to 210 cases), including several with abnormal behavior (11 to 114 cases), were nearly proportional to the increase in yearly dispensations between fiscal years 2006 and 2008 (Figure 3B). Only two deaths and one fall were reported for zanamivir in fiscal year 2008. In both age groups of children (aged less than 10 years and aged 10–19 years), the numbers of abnormal behavior reports increased in parallel with increased zanamivir dispensation between fiscal years 2006 and 2008 (Table 2). No significant differences were noted between fiscal years 2006 and 2008 in the indices of reporting trends of abnormal behaviors adjusted for the number of zanamivir dispensations in both age groups, although these reporting indices for zanamivir were consistently higher than those for oseltamivir.

## Discussion

In this retrospective review of pharmacy dispensation records and spontaneous adverse reaction reporting, we evaluated the effectiveness of actions taken in response to concerns about oseltamivir-associated risk of abnormal behavior by the regulatory authority and the influence of mass media publicity in Japan. The March 2007 DDL and subsequent package insert revision for oseltamivir advising against use in children aged 10–19 years effectively changed prescribing practices in Japan, causing a drastic, significant reduction in oseltamivir prescriptions in this age group and a substantial increase in total prescriptions of zanamivir. Reporting of abnormal behaviors associated with oseltamivir use was greatest in fiscal year 2007, partly because of heightened attention in the wake of intense adverse media coverage. Given that the decreases in the use of and abnormal behavior reports for oseltamivir in children aged 10–19 years in fiscal year 2008 were not reflected in the total number of abnormal behavior cases reported in that year, the regulatory actions targeting this age group appear to have been inappropriate in risk minimization for the entire treatment population. In contrast, we found that the number of zanamivir-associated case reports of neuropsychiatric disorders increased in proportion to the yearly increase in use. These findings will be valuable in reviewing the effectiveness and validity of current differential safety strategies for oseltamivir and zanamivir.

In Japan, nearly all patients with rapid antigen testing-confirmed influenza are prescribed antiviral medications, including oseltamivir and zanamivir [19], and a high correlation was reported between the total number of weekly pharmacy dispensations of oseltamivir and zanamivir and the number of cases reported to the sentinel influenza surveillance system [20]. The high correlation and good proportionality with nationwide influenza activity in the present study indicate the validity of pharmacy antiviral dispensation records as a national index for trends in influenza medication in Japan.

We assessed the significant, combined effects of regulatory actions, academic recommendations, and mass media publicity on changes in the prescribing practice of oseltamivir and zanamivir. Children aged 10-19 years accounted for a considerable portion of all influenza patients during the study period (25.6% in the 2006/07 season, 16.2% in the 2007/08 season, and 22.2% in the 2008/09 season) [15]. The significant decreases in oseltamivir prescribing to children aged 10-19 years after the 2007 DDL therefore evidenced good compliance with the regulatory actions; and the subsequent increase in the amount of zanamivir prescriptions in the 2008/09 season appears to have resulted from a risk-averse attitude of selecting the alternative, as suggested by a questionnaire survey of Japanese physicians [21]. Safety concerns about oseltamivir received wide and continuous coverage on the Internet and in the mass media prior to the 2007 regulatory actions, whereas zanamivir received little attention [19]. This and other adverse publicity about oseltamivir likely spurred strong changes in the prescribing behavior of practitioners, probably through heightening of public and physician sensitivity to oseltamivir risk prior to the 2007 regulatory actions. Thus, risk communication through the mass media appears to significantly influence public response to a regulatory action [22].

Although annual dispensation of oseltamivir decreased in fiscal year 2007, the number of spontaneous adverse reaction reports associated with its use was disproportionally high. At least some of these would have been due to the carryover of spontaneous case reports that were not reported at the time of occurrence in the preceding year; in fact, the domestic fiscal year 2007 Periodic Safety Reports on oseltamivir covering April 2007 to March 2008, submitted by the marketing authorization holder, listed many spontaneous adverse reaction case reports with onset dates prior to the March 2007 regulatory actions, which were received by the marketing authorization holder after April 2007 [23]. The spontaneous reporting on oseltamivir use in fiscal year 2007 should therefore be regarded as having been inflated by a number of reports with onset dates received in previous years, owing to the so-called “notoriety bias” resulting from the 2007 regulatory actions and intense adverse publicity [24].

The fact that overall spontaneous reporting on oseltamivir adverse reaction cases decreased in fiscal year 2008 indicates that the 2007 regulatory actions were likely successful in reducing the drug-associated events. The lack of reported deaths and falls in fiscal year 2008 might be ascribed to social penetration of the 2007 MHLW caution on the necessity of monitoring children with influenza within households. Nevertheless, the observed change in the total number of case reports of abnormal behavior in all oseltamivir users between fiscal years 2006 and 2008 was markedly smaller than that expected from the decrease in dispensing in children aged 10-19 years. In fact, the number of abnormal behavior reports in this age group in fiscal year 2008 was less than half that in fiscal year 2006. This decrease was, however, supplemented by an increased number of abnormal behavior reports in those aged less than ten years, implying that younger children are also at risk. The observed number of abnormal behavior cases reported in children aged less than 10 years was not negligible, which is consistent with the finding that influenza-associated encephalitis was mainly reported among younger children [25]. The oseltamivir reporting trend of abnormal behavior in children aged less than 10 years, adjusted for pharmacy dispensations, was smaller than that for children aged 10–19 years, which may be explained by either a true age effect on abnormal behavior occurrence or a potential bias caused by probably stimulated reporting for the cases in age of 10–19 years. Since the indices of reporting trends described in the present paper should be viewed as a biased surrogate of risk, cautions should be taken for their interpretation. However, the number of reports in children aged less than 10 years do not rule out the potential risk of abnormal behavior and may also raise the necessity of appropriate risk mitigation in this population. Thus, the restriction of oseltamivir use in children aged 10-19 years posed by the March 2007 regulatory actions warrants reexamination.

Our results also appear to be consistent with the assumption that the excess risk of abnormal behaviors in the oseltamivir-treated population may be ascribable to the underlying influenza, but not to oseltamivir [26]. Our results indicated that the number of abnormal behavior reports is consistently dependent on the size of populations treated with antivirals except for the reporting for oseltamivir in fiscal year 2007. Risk of neuropsychiatric symptoms associated with oseltamivir use has been predominantly indicated in case reports, but not in controlled studies and preclinical research [1], [2], [3], [27], [28], [29], [30], [31], except for one recently reported questionnaire-based study in Japan [32]. This previous study indicated a slightly elevated risk of delirium with oseltamivir use in patients under 18 during the 2006/2007-influenza season with marginal significance (hazard ratio: 1.51, 95% CI lower: 0.95, upper: 2.40, p = 0.084). Since its extensive use in the era of swine flu pandemic, no further cases of fatal falls in children in association with oseltamivir use in Japan have been reported [17].

In Japan, no strong risk minimization actions against zanamivir have been taken. However, if oseltamivir does indeed increase risk, the fact that the indices for yearly reporting trends of abnormal behavior cases for zanamivir in the present study remained constant and higher than those for oseltamivir suggests that zanamivir-treated patients also possess an excess risk of abnormal behavior. Given also that oseltamivir and zanamivir share a common pharmacological target and are prescribed in the same clinical practice, we consider that the current differential safety strategies between these two neuraminidase inhibitors should be at least aligned within the drug class.

### Limitations

The strength of inference regarding the association between drug exposures and adverse events is affected by several limitations. The source of our dispensation data was community pharmacies for outpatients with a geographically heterogeneous distribution that was not in full agreement with that of spontaneous adverse reaction reporting. Further, due to the lack of joint distribution of exposures and events at the individual level, similar to ecological studies, only weak association could be inferred in the present study [33]. Additionally, there are multiple inherent limitations in using spontaneous adverse reaction reports as outcome measurements, such as under- or delayed-reporting, diagnosis inaccuracy, lack of severity information, and inflated reporting due to intense public attention. These might bias the assessment of adverse-reaction reporting in relation to actual consumption [34]. Although there was a difference in the summary periods for dispensation for fiscal year 2006 and fiscal years 2007 and 2008, we concluded that this would not cause a substantial bias, because the dispensation of anti-influenza agents is usually concentrated in the influenza on-season, with only a relatively small portion of dispensation occurring during the influenza off-season from April to October (Figure 2). Therefore, the above discussion should be viewed as preliminary and tested with other robust methodologies, such as large-scale, prospective, controlled studies, although operational difficulties may be expected in identifying and collecting case information, such as rare abnormal behavior, with reliability and validity in a large-scale observational setting.

In conclusion, we demonstrated that the 2007 regulatory actions effectively reduced oseltamivir use in children aged 10-19 years in the wake of intense adverse publicity. This publicity would have also strongly influenced the spontaneous reporting of oseltamivir cases. Despite several important limitations of our data, our preliminary findings suggesting comparable risks among children with influenza treated with these two neuraminidase inhibitors appear to recommend reexamination of the current differential safety strategies by age and agent.

## Supporting Information

Table S1 **Summary of risk minimization actions in response to safety concerns about oseltamivir and zanamivir in Japan.**
(DOC)

Appendix S1 **Abstract translation in Japanese.**
(DOC)
