# Supplementary material for: Oseltamivir Prescription and Regulatory Actions Vis-à-Vis Abnormal Behavior Risk in Japan: Drug Utilization Study Using a Nationwide Pharmacy Database
Source: PLoS One. 2011 Dec 6;6(12):e28483. doi: 10.1371/journal.pone.0028483 (PMC3232227; doi:10.1371/journal.pone.0028483)
Supplement: Table S1 — Summary of risk minimization actions in response to safety concerns about oseltamivir and zanamivir in Japan. (DOC) [file pone.0028483.s001.doc]

## Table S1. Summary of risk minimization actions in response to safety concerns about oseltamivir and zanamivir in Japan.

| Date | Detail |
| --- | --- |
| June 2004 | Addition of neuropsychiatric symptoms to the section ‘Clinically Significant Adverse Reactions’ in the Japanese Package Insert (PI) of oseltamivir. |
| January 10, 2006 | Statement on the rational use of oseltamivir by the Japan Pediatric Society in preparation for national antiviral stockpiling for pandemic influenza. The statement requested that use be limited, for treatment use, to patients with positive results on the rapid antigen test within 48 hours of disease onset and, for preventive use, to subjects at a high risk of poor prognosis of influenza. |
| July 4, 2006 | First newspaper coverage of the death of a teenager in Japan associated with oseltamivir. |
| February 2007 | Multiple reports of cases of fatal falls after oseltamivir use by teenagers. |
| February 28, 2007 | The Japanese Ministry of Health, Labour, and Welfare (MHLW) cautioned health care professionals on the necessity of continuous household monitoring for possible abnormal behavior after influenza diagnosis regardless of oseltamivir use. |
| March 20, 2007 | After receiving two case reports of fracture due to fall in teenagers, the MHLW issued a recommendation that the ‘Precaution’ section of the oseltamivir PI be revised. A warning was added to the PI that oseltamivir was associated with a suspected but unproven risk of abnormal behavior that might lead to death due to falls and recommended that the use of oseltamivir in children aged 10-19 years be restricted. The manufacturer revised the PI and issued a ‘Dear Doctor’ letter that cautioned doctors about the risk of abnormal behavior. |
| March 25, 2007 | Statement by the Japan Pediatric Society affirming the above regulatory action and pointing out the need to monitor abnormal behavior for two days after the onset of symptoms. |
| April 13, 2007 | Recommendation by the MHLW to revise the ‘Precaution’ section of the oseltamivir PI to add that ‘oseltamivir should be used judiciously, considering that antiviral agents are not always necessary in the treatment of influenza.’ The manufacturer revised the PI accordingly. |
| December 26, 2007 | Recommendation by the MHLW to revise the ‘Important Precautions’ section of the zanamivir PI to caution health care professionals about neuropsychiatric symptoms and ask prescribers to inform parents of the risk of abnormal behavior and the need to monitor patients after the start of treatment. |
| January 27, 2008 | The Japan Pediatric Society issued a statement after receiving a report from the MHLW Subcommittee on Safety Measurements for oseltamivir held on Dec. 25, 2007. The statement re-affirmed the March 2007 regulatory action, which advised that the advisory against the use of oseltamivir in children aged 10-19 years be maintained. |
| June 16, 2009 | Announcement by the MHLW Subcommittee on Safety Measurement confirming that continuing the 2007 safety interventions was effective and appropriate, after reviewing the report of the pre-clinical and clinical working groups, which noted that the involvement of oseltamivir in abnormal behavior in children aged 10-19 years was still unclear, and that two epidemiological studies indicated a clear association of abnormal behavior with influenza. |
