# Supplementary material for: Oseltamivir Prescription and Regulatory Actions Vis-à-Vis Abnormal Behavior Risk in Japan: Drug Utilization Study Using a Nationwide Pharmacy Database
Source: PLoS One. 2011 Dec 6;6(12):e28483. doi: 10.1371/journal.pone.0028483 (PMC3232227; doi:10.1371/journal.pone.0028483)
Supplement: Appendix S1 — Abstract translation in Japanese. (DOC) [file pone.0028483.s002.doc]

**Appendix 1. Abstract translation in Japanese.**

**Title: 題目**

日本におけるオセルタミビルの処方と異常行動リスクに対する規制措置の影響：保険薬局データベースを用いた医薬品使用実態調査

**Abstract: 抄録**

**Background: 背景**

2007年3月、異常行動に関する安全性の懸念から10歳代患者におけるオセルタミビルの使用を制限するよう規制当局による指導がなされた。本規制措置の公衆衛生上の意義は重大であるにも関わらず、これまでその有効性と妥当性を検証した報告はない。本研究では、これら規制措置による処方実態と安全性報告への影響について評価を行った。

**Method and Findings: 方法及び結果**

全国展開する薬局チェーンの調剤情報データベースを用いた後ろ向き調査である。2006年11月から2009年3月までのオセルタミビルおよびザナミビルに関する100,344の調剤記録を解析した。これらの薬剤の調剤動向を、規制措置の前と後で経時的に、メディアにおけるこれら薬剤の表出頻度及び副作用自発報告数と対比させて提示した。

2007年3月の措置対策は、メディアで多く表出したこととあいまって、2006年度と同程度のインフルエンザ流行が見られた2008年度において、10歳代患者におけるオセルタミビルの調剤数を2006年度の20.4％まで減少させた。その一方で、ザナミビルの調剤数はすべての年齢層にわたって約9倍に増加した。オセルタミビルの使用に関連した10歳代患者における異常行動報告数は2006年度の24例から2008年度の9例へと減少したが、その減少は10歳未満患者での報告数増加（2006年度12例から2008年度28例）により相殺された。一方でザナミビルの使用に関連する報告数は調剤数の増加に比例して増加した（2006年度11例から2008年度114例）。

**Conclusions: 結論**

2007年の規制措置はその標的とする患者集団におけるオセルタミビルの処方と異常行動報告数を効果的に減少させた。オセルタミビル投与を受けた10歳未満患者とザナミビル投与患者における異常行動報告数の増加が観察されたことは、これらの患者集団もまたリスク集団であることを示唆する。現行の年齢別、薬剤別に異なる安全性対策の妥当性に関する検討が必要であろう。
